# Liver trauma: WSES position paper

**DOI:** 10.1186/s13017-015-0030-9

**Published:** 2015-08-25

**Authors:** Federico Coccolini, Giulia Montori, Fausto Catena, Salomone Di Saverio, Walter Biffl, Ernest E. Moore, Andrew B. Peitzman, Sandro Rizoli, Gregorio Tugnoli, Massimo Sartelli, Roberto Manfredi, Luca Ansaloni

**Affiliations:** General, Emergency and Trauma Surgery, Papa Giovanni XXIII Hospital, P.zza OMS 1, 24128 Bergamo, Italy; Emergency and Trauma Surgery, Maggiore Hospital, Parma, Italy; General, Emergency and Trauma Surgery, Maggiore Hospital, Bologna, Italy; Trauma Surgery, Denver Health, Denver, CO USA; Surgery Department, University of Pittsburgh, Pittsburgh, Pensylvania USA; Trauma & Acute Care Service, St Michael’s Hospital, Toronto, ON Canada; General and Emergency Surgery, Macerata Hospital, Macerata, Italy

**Keywords:** Liver trauma, Surgery, Hemorrage, Operative management, Non-operative management

## Abstract

The liver is the most injured organ in abdominal trauma. Road traffic crashes and antisocial, violent behavior account for the majority of liver injuries. The present position paper represents the position of the World Society of Emergency Surgery (WSES) about the management of liver injuries.

## Background

The liver is the most injured organ in abdominal trauma [1–3]. Road traffic crashes and antisocial, violent behavior account for the majority of liver injuries [2]. As demonstrated by several studies the management of liver trauma has deeply changed through the last three decades with a significant improvement in outcomes, especially in blunt trauma [1, 2, 4]. Most liver injuries are grade I, II or III and are successfully treated by observation only (Non-Operative Management, NOM). In contrast two-thirds of grade IV or V injuries necessitate laparotomy (Operative Management, OM) [3]. These operations are generally challenging and difficult. Richardson et al. proposed as the main reasons for improvement in survival: 1) improved results with packing and reoperation, 2) use of arteriography and embolization, 3) advances in operative techniques for major hepatic injuries, and 4) decrease in hepatic venous injuries undergoing operation [1, 3]. The severity of traumatic liver injuries is universally classified according to the AAST classification system (Table 1) [5]. The present paper represents the position of the World Society of Emergency Surgery (WSES) about the treatment of liver trauma. This paper results from the Second World Congress of WSES that has been held in Bergamo (Italy) on July 2013. Levels of evidence have been evaluated in agreement with the Oxford guidelines [6]. As the WSES includes surgeons from the whole world, this position paper aims to give the state of the art of the management of liver trauma, maintaining into account the secondary different possibilities in its management. In actuality, not all trauma surgeons work in the same conditions and have the same facilities and technologies.

**Table 1:** AAST organ injury scale – liver injury

Grade	Injury type	Injury description
I	Haematoma	Subcapsular < 10 % surface
	Laceration	Capsular tear < 1 cm parenchymal depth
II	Haematoma	Subcapsular 10–50 % surface area; intraparenchymal, < 10 cm diameter
	Laceration	1–3 cm parenchymal depth, < 10 cm in length
III	Haematoma	Subcapsular > 50 % surface area or expanding, ruptured subcapsular or parenchymal haematoma. Intraparenchymal haematoma > 10 cm
	Laceration	> 3 cm parenchymal depth
IV	Laceration	Parenchymal disruption 25–75 % of hepatic lobe
V	Laceration	Parenchymal disruption involving > 75 % of hepatic lobe
	Vascular	Juxtavenous hepatic injuries i.e., retrohepatic vena cav/central major hepatic veins
VI	Vascular	Hepatic avulsion

Advance one grade for multiple injuries up to grade III

AAST liver injury scale (1994 revision)

### Classification

**Fig. 1:**
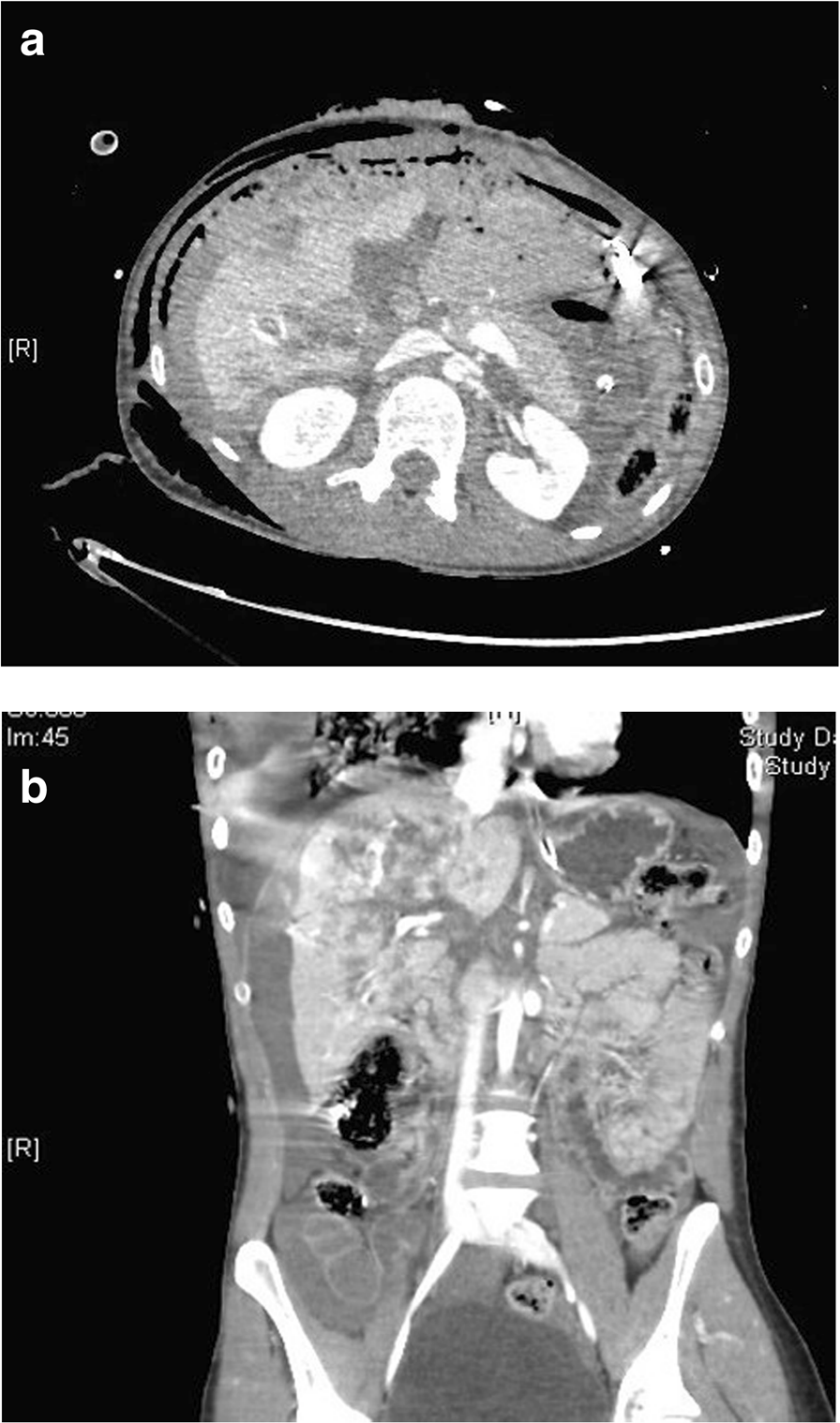
**a b** CT immages of Grade V liver injury

Hepatic traumatic lesions can be classified as minor (grade I, II), moderate (grade III) or major/severe (grade IV, V) injuries (Fig. 1a, b) [3, 7–9]. This classification is not well defined in the literature, but aims to define the type of management that can be adopted and the related outcome [8]. Frequently low-grade American Association for the Surgery of Trauma (AAST) lesions (i.e., grade I-III) are considered as minor or moderate and treated with NOM [8, 9]. However some patients with high-grade lesions (i.e., grade IV-V laceration with parenchymal disruption involving more than 75 % of the hepatic lobe or more than 3 Couinaud segments within a single lobe) may be hemodynamically stable and treated with NOM [3]. This demonstrates that the classification of liver injuries as minor or major ones must consider not only the anatomic AAST classification but more importantly, the hemodynamic status of the patient, the ISS and the associated injuries.

A few studies considered as minor injuries those lesions with hemodynamic stability, a low AAST organ injuries scale and a low ISS [8, 9]. These patients can be safely managed non-operatively with good results in term of morbidity and mortality. On the other hand major injuries are those with a higher AAST organ injuries scale, high ISS and a higher transfusions rate and are often associated with the worst outcome in terms of morbidity and mortality [8, 9]. For all the aforementioned reasons major injuries are associated with a higher necessity of OM.

### Diagnostic procedures in liver trauma (blunt and penetrating)

*Focused abdominal sonography for trauma (FAST)* has superseded the diagnostic peritoneal lavage (DPL) or diagnostic peritoneal aspirate (DPA) in many centers to evaluate the presence/absence of intra-abdominal fluid in unstable patients with blunt trauma [7]. DPL however remains valuable in patients in shock without an overt source of blood loss. The greatest advantages of FAST are that it is an economic, non-invasive, rapid, repeatable procedure, with sensitivity between 80–85 % and a specificity of 97–100 % [10]. The procedure has some limitations: reduced sensitivity and specificity in obese patients, in case of ileus, or subcutaneous emphysema, and that it is operator dependent [10]. Richards et al. [11] reported a 98 % of sensitivity in grade III to V liver injuries, but there are demonstrated differences between groups with different expertise [12]. FAST will generally document 400 ml or more of intra-peritoneal fluid, and for this reason is a useful exam in unstable patients to decide for OM or not [7]. As a counterpart if positive FAST is absolutely helpful in deciding for OM or not, an apparent negative study does not definitely exclude significant intra-peritoneal bleeding. In penetrating trauma FAST is highly specific (94.1–100 %), however is not able to evaluate the exact lesion grade and is not very sensitive (28–100 %) [7, 13].

*CT-scan* has over the last years has improved the detection of the abdominal injuries. In patients who are hemodynamically stable, with either penetrating or blunt injuries, CT is the gold standard [7, 14–16]. Triple contrast CT has been shown to have a good sensitivity, except for diaphragm, pancreas and small bowel injuries [7]. Some authors consider CT as a predictive factor, along with systolic blood pressure (SBP), to determine the risk of failure of non-operative management (NOM) and to predict the patient outcome, particularly in grade IV lesions or higher [17]. In fact, in the setting of involvement by one or more hepatic veins, liver surgery is 6.5 times more common, and there is a 3.5 times higher risk of arterial bleeding. As a counterpart, the risk of false negative for vascular injuries at CT can delay proper intervention. For this reason some authors suggested angiography in all patients with grade 3–5, irrespective of hemodynamic stability or blush on CT-scan, particularly when there is associated major hepatic venous involvement [17–19]. On the other hand hepatic angiography does not appear to be warranted in the absence of active bleeding on CT among patients with CT grade II or grade III injuries, because in these patients the principal risk appears to be venous bleeding [17].

Diagnostic peritoneal lavage (DPL) or diagnostic peritoneal aspirate (DPA) has been commonly used since its introduction in 1965. It has been the technique of choice in ATLS until being replaced by the FAST. It is a diagnostic approach to evaluate the presence of hemoperitoneum or free bowel contents in unstable patients [10]. DPL is considered rapid, accurate, and sensitive tool to identify intra-abdominal injuries, but it is an invasive procedure [10]. Contraindications for DPL are obesity, previous laparotomy, coagulopathy and advanced pregnancy [10]. Despite being replaced by FAST over the last few years, in a recent randomized controlled trial DPL was consider superior to FAST in identifying intra-abdominal injuries, even though it required significantly more time to be performed [10].

### Recommendations for Non Operative Management (NOM) in blunt liver trauma (BLT)

*Patients should undergo an initial attempt of NOM in a scenario of blunt trauma, hemodynamic stability, and isolated liver injury, irrespective of injury grade (GoR 2 A).*

*NOM is not indicated in case of hemodynamic instability or peritonitis (GoR 2 A).*

*NOM should be considered only in an environment that provides capability for patient intensive monitoring, angiography and an always available operating room (GoR 2 A).*

*Abdominal CT with intravenous contrast should be always performed to identify the liver injuries and provides critical information for consideration of NOM (GoR 2 A).*

*Angiography with embolization may be considered the first-line intervention in patients with hemodynamic stability and arterial blush on CT-scan (GoR 2 B).*

NOM for liver injury, has increased during the last century due to its high success rates (82–100 %) [14, 8, 20–28]. This non-operative approach was at first applied to pediatric patients and has rapidly been extended to adults. In blunt trauma, NOM is the standard of care in hemodynamically stable patients, without other associated injuries requiring an OM [29]. It is contraindicated in case of hemodynamic instability or peritonitis [14]. Croce et al. in a prospective case–control trial, reported a lower rate of complications and a lower number of transfusions in stable patients treated non-operatively, regardless of the liver injury severity [8].

The advantages of NOM include: lower hospital cost, earlier discharge, avoiding non-therapeutic laparotomy and unnecessary liver resection, fewer intra-abdominal complications and reduced number of transfusions [20]. However, in patients with severe head injuries and in the elderly, hypotension may be deleterious, and an OM can be suggested as safer [7].

The definition of ‘hemodynamic instability’ is not well estabilished [14]. The Advanced Trauma Life Support (ATLS) definition [30] consider as “unstable” the patient with: blood pressure < 90 mmHg and heart rate > 120 bpm, with evidence of skin vasoconstriction (cool, clammy, decreased capillary refill), altered level of consciousness and/or shortness of breath.

After hemodynamic status, the American Association for the Surgery of Trauma (AAST) grade of injury and the presence of multiple organs lesions seem to be the principal predictors of failure [31]. However there is no consensus about the NOM failure risk factors. For this reason NOM should only be attempted in centers capable of a precise diagnosis of the severity of liver injuries and capable of intensive management (frequent hemoglobin controls, frequent clinical monitoring and 24-h CT-scan, angiography and operating room availability) [20, 32–34]. At present, no studies report the optimal type and duration of monitoring. Velmahos et al. considered as predictors of NOM failure hypotension on admission, high CT-grade of injury, active contrast extravasation on CT-scan, and the need for blood transfusion [35]. Furthermore others authors add the dimension of the hemoperitoneum (blood around liver, peri-colic gutter, and in pelvis), the age greater than 55 years, the altered neurologic status, associated injuries, lactate level at the admission and drop of the hematocrit >20 % in the first hour, as risk factors for NOM failure [7, 20, 36]. However these criteria were not identified as absolute contraindications to NOM.

The total number of transfusions required, in deciding to opt either for NOM or OM, is still debated [20]. Pachter et al. suggest that more than 2 units transfusion and an intraperitoneal blood estimated quantity of more than 500 mL suggest ongoing bleeding and that an OM is necessary [37]. Carillo et al. suggested no more than 4 units of blood in hepatic-related transfusion [38], and Kozar at al. reported as predictor of liver-related complications the grade of liver injuries and the 24-h transfusion requirement [39].

To improve better use of blood products and hemostatic agents, the use of thromboelastography (TEG) and the thromboelastometry (ROTEM) analysis may be safer and helpful to guide the transfusion strategy [40]. No definitive recommendations actually exist for the use of recombination activated factor VII (rFVIIa) either in prevention or in routinely use in hemorrhage management in trauma [41]. Some authors suggest that rFVIIa has no role [42].

**Fig. 2:**
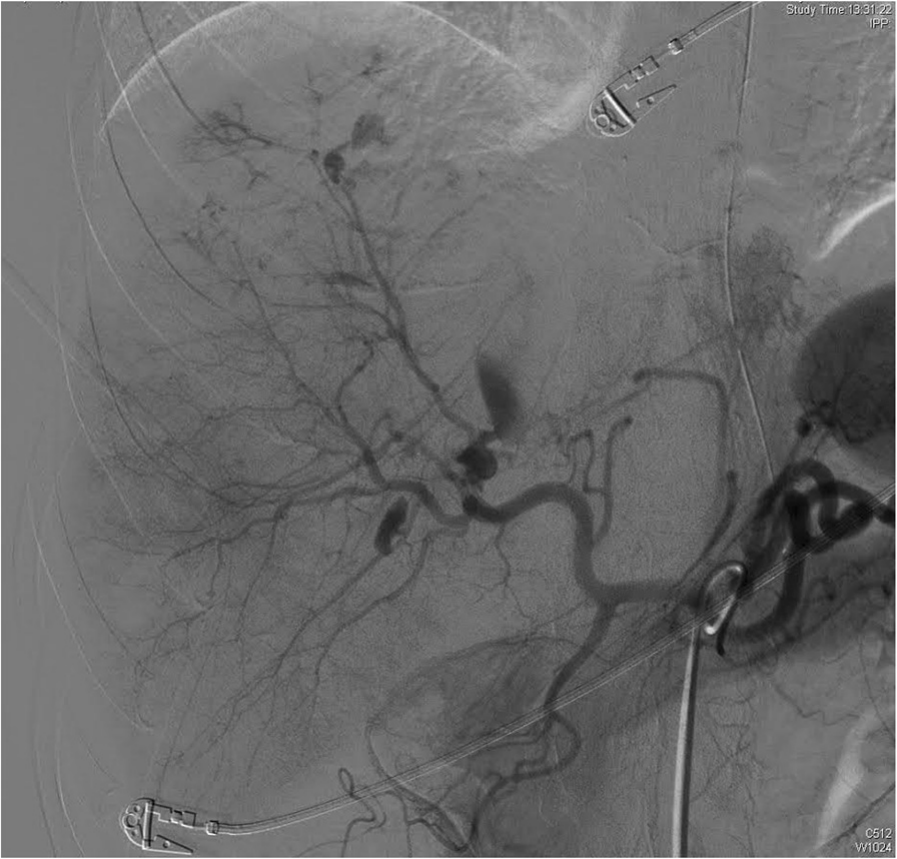
Hepatic angiography

Angioembolization is considered by several studies as an “extension” of resuscitation in patients with ongoing resuscitative needs, but this practice can be applied safely only in selected centers (Fig. 2) [14]. Some papers have reported early angio-embolization can decrease the need for transfusions and surgery [43, 44]. A recent Norwegian prospective trial with historical control, applied NOM to stable patients with blush at the CT-scan or with clinical bleeding without blush with grade 3–5 liver lesions. It demonstrated a decreased number of total laparotomy (24 % vs. 49 %) with a stable NOM failure rate (13 %), decreased transfusions and mortality, and a reduced complications rate (44 % vs. 58 %) [18]. In any case the early use of this procedure may be beneficial [31, 45].

In multi-organ injuries, particularly in cases of associated liver and splenic injuries, a recent study by Hsieh et al. reported that NOM is feasible also in case of high-grades hepato-splenic injuries (81.4 % NOM vs. 18.7 % OM) with a failure rate of 3.7 % for liver trauma and 7.1 % failure rate for the spleen trauma [46]. In multi-organ injuries predictors of failure of NOM are: initial low hemoglobin level, increased need for transfusions in ICU [46].

Complications of NOM in blunt hepatic trauma arise particularly in high-grade injury (overall complication rate: 0–7 %, complications in grade III-V injuries: 12.6 % - 14 %) [7, 14]. Clinical examination, blood tests, ultrasound and CT-scan can help in the diagnosis, but a routine follow-up with CT-scan is not necessary [3, 7, 14]. However control CT-scan is required in case of persistent inflammatory response at laboratory tests, fever, abdominal pain, jaundice and drop of hemoglobin level [14]. The most frequent complications of NOM are: biliary (bile leak, hemobilia, bilioma, biliary peritonitis, biliary fistula), bleeding, abdominal compartment syndrome, infections (abscesses and other infections) and liver necrosis [7, 20]. Ultra-sound evaluation is useful in liver trauma NOM follow-up, especially in the assessment of bile leak/biloma in grade IV-V injuries, especially with a central laceration.

The main complication that can occur is re-bleeding or secondary hemorrhage (as in the rupture of a capsulate hematoma or a pseudo-aneurysm) [7, 14]. “Late” bleedings generally occur within 72 h after trauma, and the overall incidence is 0 % to 14 %. Fortunately the majority of cases (69 %) can be treated non-operatively [7, 14]. Unlike the splenic injuries, liver lesions behave predominantly in two ways: either with a copious hemorrhage at the beginning requiring an OM, or with no active bleeding that can be safely managed with NOM [47]. Post-traumatic hepatic artery pseudo-aneurysms are rare (1.2 %, with the 70–80 % extra-hepatic and 17–25 % intra-hepatic) and they can usually be managed with selective embolization [48].

Biliary complications can occur in 1/3 of cases and can be controlled with endoscopic retrograde cholangio-pancreatography (ERCP) and eventual stenting, percutaneous drainage and lastly with surgical intervention (open or laparoscopic) [14]. Bile leaks can occur in 3–20 % of NOM [7, 14]. In case of minor bile leaks a conservative approach can be safely attempted, however high-output biliary fistula (greater than 300–400 mL/d or when bilious drainage was at least 50 mL/d continuing after 2 weeks) will benefit from an early ERCP [49]. Also intrahepatic bilio-venus fistula (frequent associated with bilemia) can be treated with ERCP [50].

Peri-hepatic abscesses have a low incidence (0 %–7 %) and can be managed with CT-scan or ultrasound-guided drainage [7, 14, 31]. Necrosis and devascularization of hepatic segments may occur and clinically may produce elevation of transaminases, coagulopathy, bile leak, abdominal pain, feeding intolerance and sepsis if more severe [7]. In these cases surgical management would be indicated [7]. Hemobilia is uncomomon (less than 3 %), but is frequently associated with pseudo-aneurysm [3, 7]. Embolization is safe and is the first approach in hemodynamically stable and non-septic patients; otherwise surgical management is mandatory [7]. Another infrequent complication is the liver compartment syndrome that may occur with the presence of large sub-capsular hematomas [7]. The decompression of the hematoma with percutaneous drainage can be safe [7]. A valid option to manage these complications could be the delayed laparotomy or laparoscopy that should be considered as a part of therapeutic strategy, and not a failure for NOM [51]. Some authors reported that delayed surgery can occur in 24 % of patients treated non-operatively, and up to 67 % in those patients with major hepatic lesions (grade IV-V) [52]. Letoublon et al. [51] considered a laparoscopic abdominal exploration between the second and fifth day safer and useful particularly in case of significant hemoperitoneum, or peritoneal inflammation or in case of any kind of clinically relevant abdominal hypertension. The simple laparoscopic or laparotomic lavage-drainage can be sufficient in the majority of the cases [51].

The trauma-related thromboembolic diseases are considered the third cause of death in patients who survive the first 24-h after trauma [53]. Deep venous thrombosis is found in 58 % of cases and the risk of pulmonary embolism ranges from 2 to 22 %. Concern of hemorrhage may delay the initiation of deep venous thrombosis prophylaxis (DVTP) in hepatic trauma is often delayed, particularly in NOM. Datta et al. in a multicenter review shows that DVTP is safe and effective if initiated within 48 h from hospital admission [54]. Also Joseph et al. confirmed data about the safety and efficacy of early DVTP in blunt solid abdominal injuries [55]. Delay in starting DVTP results in increased venous thrombo-embolic events without increasing the NOM failure rate [20, 54]. In NOM patients after liver trauma, Parks et al. [29] suggested an initial treatment with sequential compression devices and as soon as possible (when the hemoglobin level variations are ≤ 0.5 g from the previous draw) the introduction of DVTP in addition to the compression device.

The post-injury follow-up is an issue that remains unclear in NOM. There is no standard follow-up and monitoring protocol to evaluate patients with NOM liver injuries. Parks and coll. reviewed NOM guidelines for patient safety and optimal length of stay based solely on clinical criteria [29]. They suggested a serial hemoglobin measurements every 6 h for the first 24 h in stable patients with I-II grade before the discharge if patient remain stable, and every 6 h during the first 12 h and subsequently after every 12 h in grade III-IV-V injuries; the patients were allowed to walk after 24 h [29].

### Recommendations for NOM in penetrating liver trauma (PLT)

*NOM in penetrating liver trauma could be considered only in case of hemodynamic stability, absence of peritonitis and or evisceration and or impalement (GoR 2 A).*

*NOM in penetrating liver trauma should be considered only in an environment that provides capability for intensive monitoring of the patients, angiography and an operating room always viable (GoR 2 A).*

*Serial clinical examinations and local wound exploration must be always performed in case of stab wounds (GoR 2 A).*

*CT scan must be always performed to identify penetrating liver injuries suitable for NOM (GoR 2 A).*

*Angioembolisation is to be considered in case of arterial bleeding in a hemodynamic stable patient without signs of peritonitis, evisceration or impalement (GoR 2 A).*

Until past years NOM has not been considered feasible in case of penetrating trauma both in stab wounds and in gunshot wounds [7, 14, 56–62]. In fact, in these cases, the majority of surgeons considered the OM as the standard or, at least, laparoscopic exploration is considered a viable option. However, particularly for stab wounds in 70 % of patients it can be unnecessary [61]. Recent studies reviewed the conservative approach, showing a high success rate (50 % of stab wounds (SW) in the anterior abdomen and about 85 % in the posterior abdomen) [57]. This concept has been applied also in gunshot wounds (GSWs) [58]. However to decide either for NOM or for OM in these cases should be kept in mind the distinction between low and high energy penetrating trauma. Only in case of low energy, both SW and GSW, NOM can be safe. In fact high energy GSW and other ballistic injuries are perceived to be less amenable to NOM because of the high-energy transfer, and in 90 % of cases an OM is required [60, 63]. Despite that some studies reported a 25 % non-therapeutic laparotomies rate in abdominal GSWs, confirming that in selective cases NOM could be pursued [63].

10 trials and case series reported about the NOM of penetrating liver injuries with a success rate ranging from 69 % to 100 %. Some of these studies also suggested an algorithm for the management of penetrating abdominal trauma [56–60]. The key points for NOM remain: hemodynamic stability, absence of peritonitis, and an evaluable abdomen. In hemodynamic instability, in presence of peritonitis or evisceration and or impalement OM should be pursued [58–60]. These findings are particularly important in cases of gunshot injuries. Navsaria et al. suggested as predictive criteria of NOM failure in abdominal GSWs are: associated head and spinal cord injuries (that preclude regular clinical examination) and significant reduction in hemoglobin requiring more than 2–4 units of blood transfusion in 24 h [57].

The role of CT scan in the evaluation of patients with SWs has not been proven, and local wound exploration (LWE) is considered more accurate than CT-scan [58]. Some papers showed an emergency laparotomy was necessary even in presence of a negative CT-scan [59]. Biffl et al. considered CT-scan necessary particularly in NOM in obese and when the wound tract is long, tangential and difficult to determine the trajectory [59]. Particularly in case of GSWs the CT-scan can help in determining the trajectory, but not all authors consider it mandatory in all patients undergoing to NOM. Some authors did not use CT-scan at all in their algorithm, and others used CT-scan only in selected patients but without explaining selection criteria [57, 63]. Velmahos et al. reported that in GSWs the CT-scan has a specificity of 96 % and a sensibility of 90.5 % for injuries requiring laparotomy [64]. The potential benefit of CT should be to reduce the rates of non-therapeutic laparotomies and consequently to increase the patients underwent to NOM [63]. However the serial clinical examination remains the gold standard to decide for OM or NOM [63].

In case of CT scan detection of free intra- or retro-peritoneal air, free intra-peritoneal fluid in the absence of solid organ injury, localized bowel wall thickening, bullet tract close to hollow viscus with surrounding hematoma, NOM is contraindicated [56]. A strict clinical and hemoglobin evaluation should be done (4-hourly for at least 48 h, once stabilized the patient could be transferred to the ward) [57, 59, 61].

Demetriades et al. [65] reported a 27.6 % of cases in which no significant intra-abdominal injuries are found at the exploration. Thus suggests the possibility for a safe NOM in selected cases. In case of liver injuries Demetriades et al. showed a 28.8 % of patients treated non-operatively, a 24.3 % treated with simple surgical techniques, and a 22.5 % of patients treated with damage-control procedures, with an overall NOM success rate (in all organ injuries) between 60 % and 90 % [56]. In liver penetrating injuries angio-embolization may be a valuable tool to stop the hemorrhage or to treat a pseudo-aneurysm when a CT-scan blush is present [56, 57].

The main reluctance of surgeons to approach non-operatively a penetrating trauma is related to the doubt to miss others abdominal lesions, especially hollow viscus perforation [56]. However on one hand, in patients without peritonitis at the admission, no increase in mortality rates in case of missed hollow viscus perforation has been reported [66]. On the other hand non-therapeutic and routine laparotomy has been demonstrated to increase the complication rate [66]. Nevertheless OM in penetrating liver injuries has a higher liver-related complication rate (50–52 %) than in blunt ones [56].

### Follow-up after successful NOM

No definitive indications exist for post-injury follow-up and normal activity resumption in patients underwent to NOM. Some authors suggest a post discharge CT-scan and an outpatient visit after 4–6 weeks in case of grade II-V lesions [7]. In patients with uncomplicated hospital course the activity can be resumed after 3–4 months (because of the majority of lesions heal in 4 months) [7, 29]. Therefore the activity can be restarted 1 month after trauma, if the CT-scan follow-up (in grade III-V lesions) has shown a significant healing [7].

The patients have to be counseled to not remain alone for long periods and to return to the hospital immediately if they experience and increasing abdominal pain, lightheadedness, nausea or vomiting [29].

### Recommendations for Operative Management (OM) in liver trauma (blunt and penetrating)

*Patients should undergo to OM in liver trauma (blunt and penetrating) in case of hemodynamic instability, concomitant internal organs injury, evisceration or impalement (GoR 2 A)*

*Primary surgical intention should be to control the hemorrhage, to control bile leak and to allow for an intensive resuscitation as soon as possible (GoR 2 B)*

*Major hepatic resections should be avoided at first, and considered subsequently (delayed fashion) only in case of large devitalized liver portions and in centers with the necessary expertise (GoR 3 B).*

*Angioembolisation is a useful tool in case of persistent arterial bleeding (GoR 2 A).*

The leading cause of death in liver injuries is exsanguination. The decision for an OM in liver trauma mainly depends from the hemodynamics patient’s status and from the concomitant internal organ injury.

For minor (grade I-II) and moderate (grade III) liver injuries, and in favorable cases (no major bleeding at the laparotomy) minimal bleeding may be controlled by packing alone or with electrocautery, bipolar devices, or argon beam coagulation, topical hemostatic agents, omental packing [7, 9, 67, 68].

In case of severe liver injuries (grade IV-V) (Fig. 1a, b) and in not favorable cases (when the risk of “lethal triad” is high or it is already present) more aggressive procedures can be necessary (first of all hepatic manual compression and hepatic packing, with eventually vessels ligation, hepatic debridement, balloon tamponade up to shunting procedures or hepatic exclusion) associated with an intraoperative intensive resuscitation aiming to revert the lethal triad [9, 68].

In all cases of Damage Control Surgery (DCS) for liver trauma when the risk to develop abdominal compartment syndrome is high and when a second look after patients hemodynamic stabilization would be needed, a temporary abdominal closure can be safely considered [9, 67, 68].

**Fig. 3:**
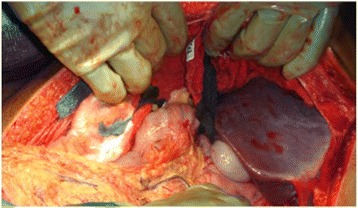
Liver packing

Hepatic packing is the first maneuver in severe hepatic injury (Fig. 3). It could be manual at first and pads compression subsequently both aiming to stop the bleeding [7, 9, 67–72]. Do not pack excessively with resultant compression of the inferior cava vein [7, 67]. Packing must be removed or changed within 48–72 h to avoid the risk of intra-abdominal sepsis [7].

**Fig. 4:**
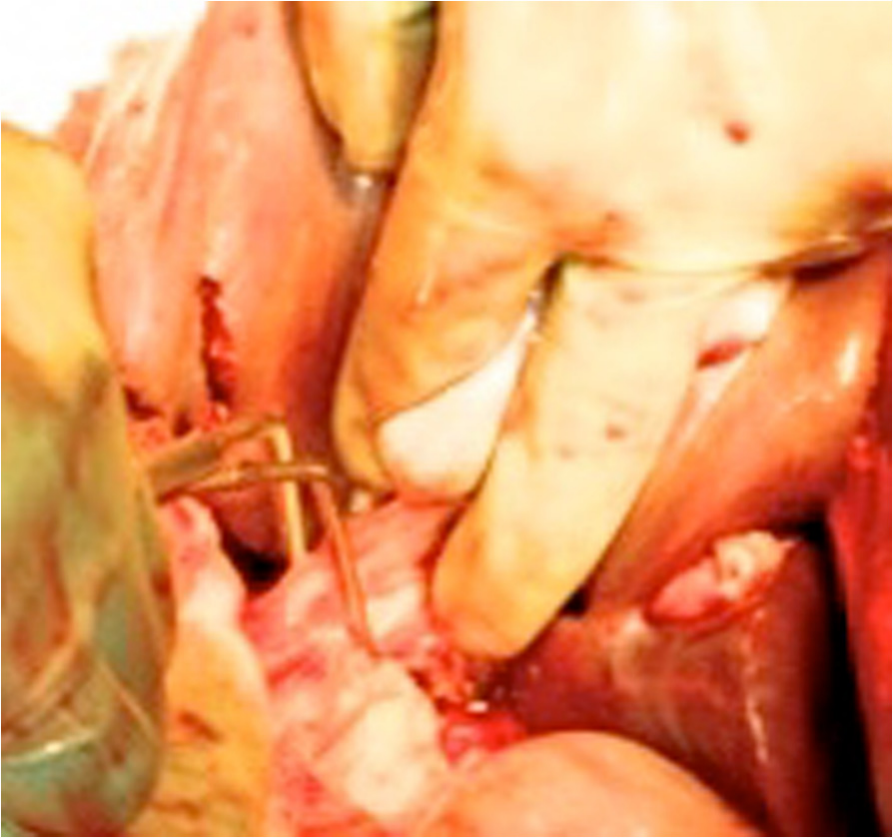
Pringle maneuver

The Pringle maneuver (with the purpose to temporarily stop the portal and arterial flow into the injured liver) is either the second option, particularly in case of persistent bleeding after hepatic packing, or to be done concurrently with packing in the patient dying of a massive liver injury (Fig. 4) (many authors advocated that clamping periods of 20 min with 5 min left for liver reperfusion decreases ischemia-reperfusion) [7, 9, 67].

**Fig. 5:**
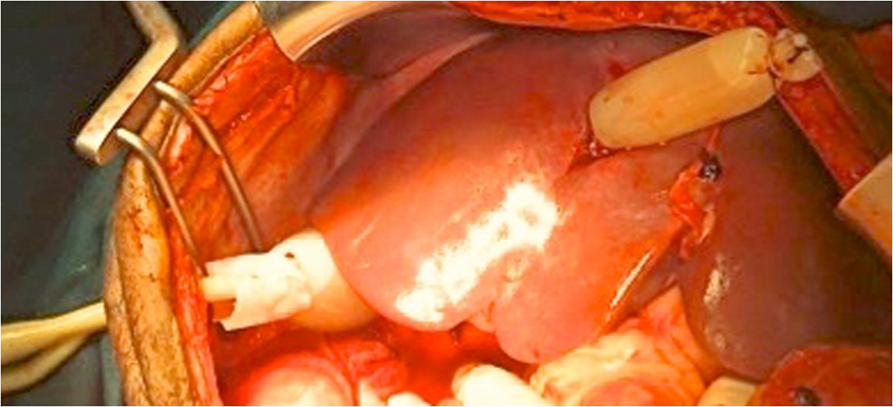
Baloon tamponade

In case of deep tracts into the liver parenchyma balloon tamponade, using a Foley or a Sengstaken-Blakemore catheter to control the hemorrhage is a viable option in patients not responding to packing alone (Fig. 5) [73]. The catheter is brought out through the skin, and can be removed after deflation 3–4 days after when the bleeding has stopped.

Fibrin sealants can be use in trauma patients and are apparently safe. These agents combine fibrin glue with thrombin, calcium chloride and aprotinin to form a stable clot [74]. However at present not many studies on human have been published, but in animal models these materials have been found to improve the bleeding control in high-grade liver lesions [75, 76].

In high-grade liver trauma, anatomic hepatic resection can be considered as a surgical option. Polanco et al. in a 15-years series of 1049 patients with liver injuries showed a decrease of mortality (9–24 % compared to 46–80 % at the beginning of the last century) and low complication rate (morbidity related to liver resection was 30 %) [3, 77, 78]. Two-thirds of 216 patients with high grade injury (with blunt and penetrating trauma) underwent surgery, and 56 underwent liver resection: 21 segmentectomies, 8 right lobectomy, 3 left lobectomies, 23 non-anatomic resections, and 1 total hepatectomy with liver transplantation. The authors reported a mortality rate from liver injury of 9 %, and an overall mortality near to 18 % [78]. However, the role of liver resection in trauma patients remains controversial and the published series demonstrate the frequency of liver resections in trauma ranges between 2 % and 5 % [3]. In unstable patients and during damage control surgery a non-anatomic resection is safer and easier [7, 9, 79]. Either anatomic or non-anatomic liver resection can be safely made with stapling device in experienced hands [79].

If bleeding persists despite the initial maneuver (hepatic packing, Pringle maneuver), and an evident hepatic artery lesion is found during operation, the artery should be repaired. If it’s impossible, a selective hepatic artery ligation can be considered as a viable option. In this case cholecystectomy (for right or common hepatic artery ligation) should be performed to avoid gallbladder necrosis [79]. This procedure is used in 1 % of patients with severe liver trauma [80]. In fact post-operative angio-embolization is a viable option, when possible, allowing hemorrhage control while reducing the complications (Fig. 2) [7, 9, 81]. In fact, after artery ligation, the risk of hepatic necrosis, biloma and abscesses increases.

Portal vein injuries should be repair primarily, and a vein ligation is to be avoided because of liver necrosis or massive bowel edema may occur. Liver Packing and a second look or liver resection are preferable to portal ligation [79].

When the Pringle maneuver or arterial control is fails to control bleeding, and bleeding persists from behind the liver, a retro-hepatic caval or hepatic vein injury is present [81]. These lesions often occur when the suspensory ligaments, diaphragm, or liver parenchyma are disrupted [7]. Therapeutic options are 3: 1) tamponade with hepatic packing, 2) direct repair (with or without vascular isolation), and 3) lobar resection [7]. Actually the most successful method of managing severe venous injuries is liver packing [7, 82–84]. Direct venous repair is less safe in non-experienced hands, with a high mortality rate [7]. However, in the past, venous repair cases with or without shunting were described. However the most of these descriptions still anecdotal; these lesions require a planned surgical intervention when suspected [7]. Pacher and Feliciano proposed direct venous repair without shunting [7]. When hepatic vascular exclusion is necessary, different types of shunting procedures have been described. The most frequent type of shunt used is the veno-veno bypass (femoral to axillary or jugular by pass) or fenestrated stent grafts by surgeons familiar with their use [7, 9, 79, 85]. The atrio-caval shunt, introduced by Schrock in 1968, by pass the retro-hepatic cava blood with a chest tube put into the inferior cava vein, up the liver, through the right atrium. Mortality rates are high, due to the complexity of the lesions and the difficulty of the procedure [9]. Liver exclusion consists to stop the blood flow to the liver and out of the liver, clamping inferior vein cave (supra-hepatic and sub-hepatic cava), the hepatic hilum (Pringle maneuver), associated or not with intra-abdominal aorta clamping [68]. This is generally poorly tolerated in the unstable patient with major blood loss.

In emergency setting, hepatic transplantation has been described in case of liver avulsion or total crush injury, when a total hepatic resection must be done. In these cases portal and systemic venous systems must be decompressed with a porto-caval shunt. During the anhepatic phase (which should last no more than 36 h) the patient will require constant intra-venous fresh-frozen plasma and glucose [79]. This procedure is also called 2-step transplantation. However the majority of patients who underwent to liver transplantation in trauma setting are transplanted during the 1^st^ week after the injury, due to liver failure in almost 50 % of cases [86]. Survivorship has been reported at 60 %.

At the moment, the exact role of post-operative angio-embolization is not well defined. Some authors reported high rate of patients who require angiography to control arterial bleeding post DCS (52–62 %) [87, 88] and others reported low mortality (12 % vs 36 %) in patients with grade IV-V hepatic injuries who underwent angio-embolization [89]. A French retrospective study has reported two principal indications in the acute post-injury phase for this procedure after high-grade liver injuries: 1) after primary operative hemostatic control in hemodynamically stable or stabilized patients, with CT-scan evidence of active bleeding, and 2) as adjunctive hemostatic control in patients with uncontrolled suspect arterial bleeding despite emergency laparotomy [90]. However not all authors agree about angiography use, and a high rate of post-procedure complications (parenchymal necrosis, bile leak, abscess and liver failure) are reported [91, 92].
